# Metacognitive Aspects of Executive Function Are Highly Associated with Social Functioning on Parent-Rated Measures in Children with Autism Spectrum Disorder

**DOI:** 10.3389/fnbeh.2017.00258

**Published:** 2018-01-10

**Authors:** Tonje Torske, Terje Nærland, Merete G. Øie, Nina Stenberg, Ole A. Andreassen

**Affiliations:** ^1^Division of Mental Health and Addiction, Vestre Viken Hospital Trust, Drammen, Norway; ^2^NevSom Department of Rare Disorders and Disabilities, Oslo University Hospital, Oslo, Norway; ^3^NORMENT, KG Jebsen Centre for Psychosis Research, University of Oslo and Oslo University Hospital, Oslo, Norway; ^4^Department of Psychology, University of Oslo, Oslo, Norway; ^5^Research Department, Innlandet Hospital Trust, Lillehammer, Norway; ^6^Division of Mental Health and Addiction, Oslo University Hospital, Oslo, Norway

**Keywords:** executive function, social function, autism spectrum disorder (ASD), behavior rating inventory of executive function, social responsiveness scale

## Abstract

Autism Spectrum Disorder (ASD) is characterized by social dysfunction. Even though executive dysfunction has been recognized as important in understanding ASD, the findings are inconsistent. This might be due to different definitions of executive function (EF), which part of EF that has been studied, structured vs. unstructured tasks, inclusion of different moderators (age, IQ, sex) and different diagnostic categories within the spectrum. The main finding is that people with ASD have more EF difficulties than normal controls and more difficulties on open-end tasks than on structured cognitive tasks. Since some EF difficulties may not be observable in a laboratory setting, informant measures might have higher ecological validity than neuropsychological tests. Evidence suggests that executive dysfunctions are associated with social impairments, but few studies have investigated the details of this relationship, and it remains unclear what types of EF deficits are relevant for the social problems of individuals with ASD. Here we investigated which EF domains were associated with various domains of social function on parent-rated measures. A total of 86 children and adolescents with a diagnosis of ASD were included and tested for general cognitive abilities. Parents completed the Behavior Rating Inventory of Executive Function (BRIEF) and the Social Responsiveness Scale (SRS). Multiple regression analysis revealed significant associations between SRS scores and age, sex, total IQ and the BRIEF indexes. The Metacognition Index from the BRIEF added significantly to the prediction of the SRS total score and the subscales Social Communication, Social Motivation and Autistic Mannerisms. The findings suggest that metacognitive aspects of EF are of particular importance for social abilities in children and adolescents with ASD. Earlier research has shown that typically developing (TD) children have a different relationship between EF and social function than children with ASD. They found that in TD children the EF domain related to behavioral regulation was most important to social function. The results from the current study may have implications for understanding the cognitive components of the social problems that define ASD, and may be relevant in developing more targeted clinical EF interventions related to core ASD dysfunctions.

## Introduction

Autism Spectrum Disorder (ASD) is characterized with persistent deficits in social communication and social interaction across multiple contexts, and restricted, repetitive patterns of behavior, interests, or activities (American Psychiatric Association, 2013). Executive function (EF) deficits are common in children with ASD (Pennington and Ozonoff, 1996; Hill, 2004; Geurts et al., 2014a), but not part of the diagnostic criteria. Furthermore, EF correlates strongly with adaptive behavior (Gilotty et al., 2002) and influences Quality of Life (QoL) in children with ASD (de Vries and Geurts, 2015). EF is often defined as the process of physical, cognitive, and emotional self-control and self-regulation that are necessary to maintain an effective goal-directed behavior (Pennington and Ozonoff, 1996; Corbett et al., 2009; Diamond, 2013). EF comprises several components including inhibition, working memory, flexibility, emotional control, initiation, planning, organization, and self-control (Miyake et al., 2000; Hill, 2004). Even though executive dysfunction has been recognized as important in understanding ASD, the findings are inconsistent (Van Eylen et al., 2015). One explanation might be that EF is an umbrella term comprising several components, and researchers have focused on different subdomains. Meta-analyses and reviews have been written about domains like inhibition and interference control (Geurts et al., 2014b), cognitive flexibility (Leung and Zakzanis, 2014) and working memory in ASD (Barendse et al., 2013; Wang et al., 2017). All conclude that patients with ASD have EF deficits within the investigated areas, but not all found differences between ASD and normal controls on neuropsychological testing. Furthermore, the most consistent and striking difficulties are seen on tasks that are open-ended in structure, lack explicit instructions and involve arbitrary rules (White, 2013; Van Eylen et al., 2015). Therefore, some of the inconsistency is suggested to be due to different types of measurements (parent-rated measures vs. neuropsychological testing). Individuals with ASD often display pronounced EF deficits in daily life, while performing adequately on highly structured neuropsychological tasks (Kenworthy et al., 2008). The presence of comorbidities like Attention Deficit Hyperactivity Disorder (ADHD) also increase the risk of EF difficulties (Craig et al., 2016). Most research has focused on data from neuropsychological assessments of EF and/or how EF impairment is related to a diagnosis of ASD (Hill, 2004; Leung et al., 2015). Since some EF difficulties may not be observable in a laboratory setting, informant measures might have higher ecological validity than neuropsychological tests (Kenworthy et al., 2008). For this reason, questionnaires have been developed to investigate EF deficits in everyday life settings (Gioia et al., 2000). A frequently used scale is the Behavior Rating Inventory of Executive Function (BRIEF) (Gioia et al., 2000). The BRIEF is divided into a Behavioral Regulation Index (BRI), and a Metacognition Index (MI), which together form a Global Executive Composite (GEC). The BRI comprises the child's ability to modulate both behavior and emotional control, and the ability to move flexible from one activity to another. The MI is related to the child's ability for active problem solving, and to initiate, organize and monitor their own actions (Gioia et al., 2000). Deficits in metacognitive aspects of EF (MI) have in earlier studies shown to be of particular importance to adaptive functioning in high functioning children with ASD (Gilotty et al., 2002).

While both social and EF deficits in ASD have been extensively studied separately, there has been limited research on the *relationship between* EF and social function. Some findings underpin the relationship between EF and key social concepts in ASD like *joint attention* and *Theory of Mind* (*ToM*). Dawson (Dawson et al., 2002) argues that performance on ventromedial prefrontal EF tasks is strongly correlated to joint attention ability in young children. Joint attention is an important prerequisite for social functioning and often impaired in ASD (Dawson et al., 2002). Pellicano found that individual differences in EF in early life, predicted change in children's ToM skills (Pellicano, 2010). This line of research has to a large extent been based on laboratory measures and neuropsychological test results. Leung et al. explored the role of EF in social impairment in ASD using informant-based measures (Leung et al., 2015). They reported that both BRI and MI from the BRIEF predicted social functioning in children with ASD, while only BRI predicted social functioning in the general population. In contrast, Kenworthy et al. (2009) found that EF, such as behavior regulation from BRIEF and semantic fluency and divided auditory attention, correlated with autistic symptoms. In an objective neuropsychological assessment of EF in ASD children, Landa and Goldberg (2005) found no relationship between EF and social skills.

Due to the heterogeneity of ASD (Lai et al., 2014), more insight may also be gained from studying the range of social difficulties beyond diagnostic categories. The Social Responsiveness Scale (SRS) (Constantino and Gruber, 2005), is a questionnaire designed to identify the presence and severity of social impairments associated with ASD. The SRS consists of five subscales which were developed to differentiate between the subcategories of social impairments for children with ASD (Constantino and Gruber, 2005). This continuous scale allows trait quantification and focuses on functions closer linked to activities in everyday life that diagnostic tools might fail to capture (Achenbach, 2011; Nelson et al., 2016). Most findings support a one-factor model of SRS (Constantino et al., 2004; Bolte et al., 2008), while others have found evidence for several dimensions (Nelson et al., 2016). Although most research with the SRS has focused on the total score only, knowledge about how the different subscales are related to EF will provide new and more differentiated knowledge about the social deficiencies in ASD and may have important implications for interventions.

Furthermore, it remains unclear what types of EF deficits are most relevant for the social problems of children and adolescents with ASD. It is known that the intelligence quotient (IQ), age and sex are factors that might influence the EF in children with ASD, supported by findings of increasing EF deficits with age (Rosenthal et al., 2013), sex-dependent EF deficits (Bolte et al., 2011; Lemon et al., 2011; Lehnhardt et al., 2016) and a relationship between EF and intelligence (Diamond, 2013; Blijd-Hoogewys et al., 2014; Rommelse et al., 2015). Thus, the degree to which IQ, sex, and age influence the relationship between EF and social problems needs to be clarified.

There is an increased interest in possible sex differences in ASD, since there might be important biological and behavioral differences between girls and boys with ASD (Halladay et al., 2015; Lai et al., 2015). However, there are inconsistent findings regarding differences in the composition of EF difficulties in girls and boys with ASD. Some have found that girls have more EF difficulties with inhibition (Lemon et al., 2011), while others reported that girls outperform boys on EF tasks related to processing speed and verbal fluency (Bolte et al., 2011; Lehnhardt et al., 2016). White et al. (2017), showed that girls with ASD have more problems related to everyday EF than boys. To the best of our knowledge, there are no studies of possible sex differences in the relationship between everyday EF deficits and social function related to ASD symptoms. However, Bolte et al. (2011) found a correlation between EF difficulties on performance based tasks and stereotypical behaviors in boys.

A review showed that EF continues to develop throughout childhood in typical developed children, reaches adult-like levels in mid-adolescence, and that the different EF components vary in their developmental trajectories (Best and Miller, 2010). Van den Bergh et al. (2014) found that inhibition problems in everyday life were more pronounced in young ASD children, and that planning was more evident for the oldest group. Contrary to their expectations they did not find a relationship between ASD severity and EF (Van den Bergh et al., 2014). In a longitudinal study by Andersen et al. (2015a) maturation of inhibition and cognitive flexibility from childhood to adolescence was found in ASD, even though the ASD group was more impaired in EF than typically developing (TD) children. They concluded that there may be a delayed development of EF in ASD, and suggested a possible developmental arrest for working memory (Andersen et al., 2015b). Rosenthal et al. (2013) on the other hand showed that there were widening divergences with age between children with ASD and TC on EF tasks in everyday life, especially in metacognitive executive abilities. They also reported significant and quite stable problems with flexibility in ASD (Rosenthal et al., 2013).

The aim of the present study was to explore the relationship between social functioning measured with the SRS (total score and the subscales Social Awareness, Social Cognition, Social Communication, Social Motivation and Autistic Mannerisms) and everyday EF measured with the BRIEF (the indexes BRI and MI) in a clinical sample of children with ASD. We also investigated potential sex differences by comparing girls and boys, and possible age differences by splitting the sample at 12 years of age. We hypothesized that there is a significant positive association between parental reports of SRS scores and BRIEF scores in children with ASD, controlling for age, IQ and sex. Furthermore, we hypothesized that both BRI and MI from the BRIEF are significant predictors of the SRS total score.

## Methods

### Participants

The study was part of the national BUPgen network, recruiting patients from Norwegian health services specializing in the assessment of ASD and other neurodevelopmental disorders. The current sample comprised 86 children with ASD, recruited between 2013 and 2016 and assessed at age 6–18 years. Thirteen of the children (15.1%) had childhood autism, one had atypical autism (1.2%), 41 (47.7%) had Asperger syndrome and 31 (36%) had unspecified pervasive developmental disorder (PDD-NOS) (Table 1).

**Table 1:** Child characteristic.

	**Total (*n* = 86)**		**Girls (*n* = 23)**	**Boys (*n* = 63)**	***p*-value**	**6–12 years (*n* = 36)**	**13–18 years (*n* = 50)**	***p*-value**
Girls/boys	23/63		23	63		11/25	12/38	0.622^a^
Childhood autism (F84.0)	13 (15.1%)		1 (4.3%)	12 (19.0%)	n/a	7 (19.4%)	6 (12.0%)	0.462^a^
Atypical autism (F84.1)	1 (1.2%)		1 (4.3%)	0	n/a	1 (2.8%)	0	n/a
Asperger syndrome (F84.5)	41 (47.7%)		12 (52.2%)	29 (47.7%)	0.142^a^	17 (47.2%)	24 (48.0%)	0.462^a^
PDD-NOS^b^ (F84.9)	31 (36.0%)		9 (39.1%)	22 (34.9%)	0.142^a^	11 (30.6%)	20 (40.0%)	0.462^a^
Comorbid ADHD^c^	28 (32.6%)		6 (26.1%)	22 (34.9%)	0.604^a^	11 (30.6%)	17 (34.0%)	0.818^a^
	**Mean (*****SD*****)**	**Range**	**Mean (*****SD*****)**	**Mean (*****SD*****)**		**Mean (*****SD*****)**	**Mean (*****SD*****)**	
Age	13.0 (2.7)	6–18	13.4 (2.4)	12.9 (2.9)	0.417	10.5 (1.7)	14.9 (1.7)	<0.001^*^
Total IQ^d^	93.4 (14.5)	71–133	89.8 (10.8)	94.7 (15.5)	0.164	93.7 (12.0)	93.1 (16.2)	0.867
Verbal IQ	91.2 (17.9)	58–134	87.4 (15.7)	92.7 (18.6)	0.231	89.9 (15.0)	92.2 (19.9)	0.555
Performance IQ	105.3 (16.3)	59–142	100.5 (11.2)	107.1 (17.5)	0.097	107.2 (15.0)	104.0 (17.1)	0.364

* *p < 0.01*.

*n/a, Not Applicable*.

a *Chi-square*.

b *PDD-NOS, Pervasive developmental disorder unspecified*.

c *ADHD, Attention deficit/hyperactivity disorder*.

d *IQ, Intelligence Quotient*.

Male: female ratio was 2.7:1. In total, 42 children were diagnosed with at least one comorbid disorder. Attention deficit/hyperactivity disorder (ADHD) was the most common comorbid diagnosis, and 28 participants (32.6%) had an ADHD diagnosis in combination with their ASD diagnosis. Furthermore, we divided our sample into; girls and boys, and two age groups above and below 12 years (6–12 years and 13–18 years). Our sample included 23 girls and 63 boys. There were no significant differences between girls and boys on age, IQ or proportion of comorbid ADHD. More boys had a diagnosis of childhood autism, but there were no significant differences between the sexes in the distribution of the Asperger syndrome or PDD-NOS diagnoses. There were 36 children in the youngest age group and 50 adolescents in the oldest group. In these two age groups there were no significant differences in sex distribution, IQ, proportion of ADHD or ASD diagnoses (Table 1). All participants had an IQ within the normal range based on a standardized Wechsler's test (Total IQ ≥ 70) and spoke Norwegian fluently. The participants total IQ fell within 2 standard deviations of the average normal score when including confidence intervals. Exclusion criteria were significant sensory losses (vision and/or hearing).

### Clinical assessment

The children were assessed by a team of experienced clinicians (clinical psychologists and/or child psychiatrists). Diagnostic conclusions were best-estimate clinical diagnoses derived from tests, interview results and observations. All diagnoses were based on the International Statistical Classification of Diseases and Related Health Problems 10th Revision (ICD-10) (World Health Organization, 1992) criteria, and the autistic symptoms were evaluated using the Autism Diagnostic Observation Schedule (ADOS) (Lord et al., 2000) and/or Autism Diagnostic Interview-Revised (ADI-R) (Rutter et al., 2003b) and/or the Social Communication Questionnaire (SCQ) (Rutter et al., 2003a). In addition, the assessment included a full medical and developmental history, physical examination, and IQ assessment. The current study included children with ASD and comorbid ADHD. Results from recent studies indicate that neuropsychiatric disorders overlap with respect to both symptoms and causes (Moreno-De-Luca et al., 2013), and it has been recognized by both clinicians and researchers for some time that ASD and ADHD often co-occur (Yerys et al., 2009). In the DSM-5, other behavioral diagnosis may accompany a diagnosis of ASD, for example ADHD (American Psychiatric Association, 2013).

### Measures

#### Social function

The parent version of the SRS (developed for the age group 4–18 years) (Constantino and Gruber, 2005) was used to identify social communication difficulties. The SRS is used in screening and/or as an aid to a clinical diagnosis of ASD and is comprised of 65 questions, rated on a 4-point Likert scale. In addition to a total score, the SRS consists of five subscales: Social Awareness, Social Cognition, Social Communication, Social Motivation and Autistic Mannerisms. The SRS has been translated into Norwegian (Ørbeck, 2009). Internal consistency is high, both in population based samples and clinical samples (Cronbach's alpha = 0.93–0.97) (Constantino and Gruber, 2005). The SRS's reliability and validity have proven satisfactory in both population based and clinical samples in Europe and in USA (Bolte et al., 2008; Wigham et al., 2012), and correlate well with Autism Diagnostic Interview-Revised (ADI-R) scores (Constantino et al., 2003). *T-scores* of ≥76 are strongly associated with a clinical diagnosis of Autistic Disorder, Asperger's Disorder, or more severe cases of pervasive developmental disorder not otherwise specified (PDD-NOS). *T-scores* of 60–75 represent mild to moderate deficits in reciprocal social behavior that is clinically significant, resulting in mild to moderate interference in everyday social interactions.

#### Executive function (EF)

In order to assess EF the parents completed the parent version of the BRIEF (Gioia et al., 2000). The BRIEF for children and adolescents aged 5–18 years includes 86-item parent and teacher forms that allow professionals to assess everyday EFs in the home and school environments (Gioia et al., 2000). The BRIEF contains eight clinical scales that are grouped in a Behavioral Regulation Index (BRI): Inhibit, Shift and Emotional Control, and a Metacognition Index (MI): Initiate, Working Memory, Plan/Organize, Organization of Materials and Monitor. *T-scores* of ≥65 are considered to represent clinically significant areas. The Global Executive Composite (GEC), is a summary score that incorporates all eight clinical scales. The GEC has high reliability in both standardized and clinical samples (Cronbach's alpha = 0.80–0.98). The current study used the Norwegian version of the parent rating form, which has high internal consistency (Cronbach's alpha = 0.76–0.92) (Fallmyr and Egeland, 2011). Similar levels are reported for the English version (Cronbach's alpha = 0.80–0.98) (Gioia et al., 2000).

#### Intelligence quotient (IQ)

IQ was assessed using age-appropriate Wechsler tests of intelligence (Wechsler, 2002, 2003, 2008).

### Statistical analyses

Data analyses were conducted using the statistical package IBM SPSS Statistics for Windows, version 23.0 (SPSS, Inc., Chicago, Illinois). Descriptive analyses and bivariate correlations were conducted. Pearson's independent *t*-test was used to compare means between girls and boys, and the two age groups. Chi-square for crosstabs was used to investigate differences in the distribution of autism diagnoses and comorbid ADHD. The differences between the subgroups correlation coefficients were calculated using http://vassarstats.net/rdiff.html (two-tailed). Because of small subgroup sizes and no significant differences between the correlation coefficients, the regression analyses were done on the total sample and sex and age were incorporated as independent variables. The assumptions of linearity, multicollinearity, independence of errors, homoscedasticity, unusual points and normality of residuals were met. Separate multiple regression analyses were conducted to explain the variance in SRS scores (total and subscales) from age, sex, total IQ and BRIEF (BRI and MI subscales). Bonferroni correction was used to correct significance level for multiple comparisons on all the analyses in this paper, and the *p* < 0.01 level (*p* < 0.05/5 = *p* < 0.01) is used in all the regression analyses. The *p* < 0.003 level is used for the correlation analyses (*p* < 0.05/18 = *p* < 0.003). For all the other analyses (*t*-tests, Chi-square and difference between correlation coefficients) we used *p* < 0.01.

### Ethical considerations

The study was approved by the Regional Ethical Committee and the Norwegian Data Inspectorate (REK #2012/1967), and was conducted in accordance with the Helsinki Declaration of the World Medical Association Assembly. The study is based on tests that are included in regular clinical assessment. The patient, the family and the professional network around them were offered information about the test results, the diagnostic process and recommended interventions. Written informed consent was obtained from all the individual participants included in the study.

## Results

### SRS and BRIEF scores

The mean SRS total score was in the severe range (*T-score* = 78.5). The highest mean score was on the Social Mannerisms subscale (*T-score* = 80.3). The lowest mean score was on the Social Awareness subscale (*T-score* = 65.3). Means and standard deviations for SRS total score and treatment subscales are shown in Table 2.

**Table 2:** *T-scores* for the Social Responsiveness Scale (SRS) and the Behavior Rating Inventory of Executive Function (BRIEF).

	**Total (*n* = 86)**	**Girls (*n* = 23)**	**Boys (*n* = 63)**	***p-*value**^**a**^	**6–12 years**	**13–18 years**	***p*-value**^**a**^
	**Mean (*SD*)**	**Mean (*SD*)**	**Mean (*SD*)**		**Mean (*SD*)**	**Mean (*SD*)**	
**SOCIAL RESPONSIVENESS SCALE (SRS)**							
SRS total	78.5 (15.0)	85.1 (16.4)	76.1 (13.8)	0.013	78.7 (14.6)	78.4 (15.4)	0.929
Social awareness	65.3 (13.4)	68.9 (15.3)	64.0 (12.5)	0.135	68.9 (10.9)	62.8 (14.6)	0.036
Social cognition	74.3 (16.6)	81.6 (19.8)	71.6 (14.5)	0.013	73.1 (17.0)	75.1 (16.4)	0.591
Social communication	73.9 (13.8)	79.2 (14.9)	72.0 (13.0)	0.032	75.0 (13.5)	73.2 (14.1)	0.551
Social motivation	78.4 (13.0)	82.8 (10.3)	76.8 (13.6)	0.060	76.7 (12.5)	79.7 (13.3)	0.288
Social mannerisms	80.3 (17.5)	86.0 (19.4)	78.2 (16.4)	0.068	79.4 (18.3)	80.9 (17.1)	0.714
**BEHAVIOR RATING INVENTORY OF EXECUTIVE FUNCTION (BRIEF)**							
Global executive composite (GEC)	67.1 (11.3)	67.9 (10.1)	66.8 (11.8)	0.707	66.7 (12.7)	67.4 (10.4)	0.792
Behavioral regulation index (BRI)	68.5 (13.5)	66.1 (13.1)	69.3 (13.7)	0.328	68.0 (14.2)	68.8 (13.1)	0.801
Metacognition index (MI)	64.4 (10.3)	67.3 (9.0)	63.3 (10.6)	0.107	64.0 (11.6)	64.6 (9.4)	0.795
Inhibit	63.0 (14.5)	62.1 (15.2)	63.3 (14.4)	0.749	62.6 (13.9)	63.2 (15.1)	0.838
Shift	72.2 (13.9)	67.6 (12.8)	73.8 (13.9)	0.060	71.4 (14.5)	72.7 (13.5)	0.668
Emotional control	64.7 (12.7)	62.9 (13.2)	65.4 (12.5)	0.433	64.3 (13.8)	65.0 (11.9)	0.823
Initiate	62.8 (11.1)	63.8 (10.3)	62.4 (11.5)	0.598	60.7 (10.4)	64.2 (11.5)	0.150
Working memory	67.2 (10.6)	68.6 (10.0)	66.7 (10.9)	0.460	67.2 (10.4)	67.2 (10.9)	0.982
Plan/organize	63.7 (11.0)	67.9 (10.4)	62.2 (11.0)	0.035	63.2 (12.6)	64.1 (9.9)	0.695
Organization of materials	54.7 (11.1)	58.5 (9.5)	53.4 (11.3)	0.057	54.3 (12.1)	55.0 (10.4)	0.778
Monitor	62.3 (12.2)	63.1 (12.3)	62.1 (12.2)	0.736	62.6 (12.8)	62.2 (11.9)	0.867

^*^p < 0.01.

a *Independent t-tests were conducted for comparisons between girls and boys, and between the age groups 6–12 years and 13–18 years*.

*Elevated SRS T-scores indicate a high degree of impairment. T-scores of 76 or higher are strongly associated with a clinical diagnosis of ASD. T-scores of 60–75 indicate deficiencies in reciprocal social behavior that are clinically significant and are resulting in mild to moderate interference in everyday social interactions*.

*Elevated BRIEF T-scores indicate a higher degree of impairment, with T-scores of 65 and above considered to represent clinically significant areas*.

The results from the BRIEF were in the clinically significant range on the indexes BRI (*T-score* = 68.5) and GEC (*T-score* = 67.1). The mean MI score from the BRIEF was *T-score* = 64.4. The highest mean score was found on the subscale Shift (*T-score* = 72.2), and the lowest mean score was found on the subscale Organization of Materials (*T-score* = 54.7). All other subscales had *T-score* averages between 62 and 67 (Table 2).

There were no significant differences between girls and boys on BRIEF and SRS scores. However, there was a strong tendency for girls to have higher scores (more problems) on SRS total (*p* = 0.013) and Social Cognition (*p* = 0.013), and a tendency for higher scores on the subscales Social Communication (*p* = 0.032) and Plan/Organize (*p* = 0.035). There were no significant differences between the two age groups on BRIEF and SRS scores. However, there was a tendency for the youngest group to have higher scores (more problems) on the Social Awareness subscale from the SRS (*p* = 0.036).

### The relationship between the SRS and the BRIEF

There was a statistically significant (*p* < 0.001–0.005) relationship between all the subscales on the SRS and the BRIEF indexes, except the SRS subscale Social Motivation and the BRIEF index BRI. The proportion of shared variance (*r*^2^) varied between 6 and 37%. The strongest positive correlation was found between the SRS total score and the BRIEF index scores GEC (*r* = 0.62, *p* < 0.001) and MI (*r* = 0.60, *p* < 0.001). There was also a moderate positive correlation between the SRS total score and BRI (*r* = 0.48, *p* < 0.001). For Pearson's correlation coefficients, see Table 3A.

**Table 3A:** Associations between social function (SRS) and executive function (BRIEF) assessed with questionnaires (*N* = 86).

		**Social Responsiveness Scale (SRS)**					
		**SRS**	**Social**	**Social**	**Social**	**Social**	**Autistic**
		**total**	**awareness**	**cognition**	**communication**	**motivation**	**mannerisms**
BRIEF Behavioral regulation index	Pearson r	0.48^*^	0.33^*^	0.46^*^	0.47^*^	0.24	0.47^*^
	*p*-value	<0.001	0.002	<0.001	<0.001	0.025	<0.001
BRIEF Metacognition index	Pearson r	0.60^*^	0.36^*^	0.54^*^	0.55^*^	0.46^*^	0.57^*^
	*p*-value	<0.001	0.001	<0.001	<0.001	<0.001	<0.001
BRIEF Global executive composite	Pearson r	0.61^*^	0.39^*^	0.56^*^	0.57^*^	0.41^*^	0.58^*^
	*p*-value	<0.001	<0.001	<0.001	<0.001	<0.001	<0.001

* *Significance after correction for multiple testing is set to p < 0.003 (2-tailed)*.

*BRIEF, Behavior Rating Inventory of Executive Function*.

Girls generally had a stronger relationship between the SRS total and the BRIEF index scores than boys (see Table 3B). However, the differences between the correlation coefficients for girls and boys were not significant, calculated using http://vassarstats.net/rdiff.html (to-tailed) (*p* = 0.197–0.390). For girls there was a strong and significant relationship between the SRS total and all the BRIEF indexes. The proportion of shared variance (*r*^2^) for the correlations for girls was 41–58%. For boys there was a moderate to strong relationship between the SRS total and the BRIEF index scores, and all the correlations were significant. The proportion of shared variance (*r*^2^) for the correlations for boys was 24–34%.

**Table 3B:** Associations between social function (SRS) and executive function (BRIEF) assessed with questionnaires for the subgroups girls and boys, and the age groups 6–12 years and 13–18 years.

		**SRS total**		**SRS total**	
		**Girls**	**Boys**	**6–12 years**	**13–18 years**
		***n* = 23**	***n* = 63**	***n* = 36**	***n* = 50**
BRIEF Behavioral regulation index	Pearson r	0.64^*^	0.49^*^	0.67^*^	0.34
	*p*-value	0.001	<0.001	<0.001	0.015
BRIEF Metacognition index	Pearson r	0.72^*^	0.54^*^	0.77^*^	0.47^*^
	*p*-value	<0.001	<0.001	<0.001	0.001
BRIEF Global executive composite	Pearson r	0.76^*^	0.58^*^	0.78^*^	0.48^*^
	*p*-value	<0.001	<0.001	<0.001	<0.001

* *Significance after correction for multiple testing is set to p < 0.003 (2-tailed)*.

*SRS, Social Responsiveness Scale*.

*BRIEF, Behavior Rating Inventory of Executive Function*.

For the youngest age group (6–12 years) there was a strong relationship between the SRS total and the BRIEF index scores, and all the relationships were significant. For the oldest group (13–18 years) the relationship between BRI and SRS total was not significant. However, the MI and GEC showed significant relationships to the SRS total. The proportion of shared variance (*r*^2^) for the correlations was 45–61% for the youngest group and 12–23% for the oldest group (see Table 3B). The differences between the correlation coefficients between the youngest and the oldest group were not significant, but showed strong tendencies in the relationship between SRS total and GEC (*p* = 0.021), between SRS total and MI (*p* = 0.024) and SRS total and BRI (*p* = 0.044).

A multiple regression analysis was conducted to identify the relations between SRS total score and age, sex, total IQ, BRIEF BRI and BRIEF MI. This model statistically significantly explained SRS total; *F*_(5, 80)_ = 12.57, *p* < 0.001, *R*^2^ = 0.440. Only BRIEF MI had a significant independent contribution to the prediction, *p* = 0.001. This result remained when the children with comorbid ADHD were removed from the analysis. For the children with ASD without ADHD (*n* = 58), the regression model with age, sex, total IQ, BRIEF BRI, and BRIEF MI significantly explained SRS total; *F*_(5, 52)_ = 10.31, *p* < 0.001, *R*^2^ = 0.498. Only BRIEF MI had a significant independent contribution to the prediction, *p* = 0.003. Regression analyses were also done with each SRS subscale as dependent variables on the total sample (*n* = 86), and all the regression models were significant (*p* < 0.001). For the subscales Social Communication, Social Motivation and Social Mannerisms, only MI from the BRIEF had a significant independent contribution to the predictions. For the subscale Social Awareness, only age had a significant independent contribution to the prediction (*p* = 0.001). None of the independent variables made an independent contribution on the subscale Social Cognition, but both sex and MI showed strong tendencies (*p* = 0.012 and 0.016). The details are described in Table 4.

**Table 4:** Regression models summary (*N* = 86).

**Factors**	**Social awareness**			**Social cognition**			**Social communication**			**Social motivation**			**Social mannerism**			**SRS Total**		
	**B**	**Sig**.	**95%**	**B**	**Sig**.	**95%**	**B**	**Sig**.	**95%**	**B**	**Sig**.	**95%**	**B**	**Sig**.	**95%**	**B**	**Sig**.	**95%**
Constant	38.37	0.010	9.40/67.35	16.78	0.322	−16.72/50.28	32.92	0.019	5.55/60.29	49.31	0.001	20.64/77.97	20.39	0.253	−14.86/55.64	26.83	0.065	−1.69/55.36
Sex	5.15	0.096	−0.94/11.24	9.08	0.012	2.04/16.11	6.42	0.029	0.66/12.17	2.94	0.334	−3.08/8.97	5.99	0.112	−1.42/13.39	7.50	0.015	1.51/13.49
Age	−1.59	0.001^*^	−2.54/−0.65	−0.51	0.354	−1.60/0.58	−0.94	0.038	−1.83/−0.05	0.31	0.511	−0.62/1.24	−0.53	0.358	−1.68/0.61	−0.80	0.089	−1.73/0.13
Total IQ	0.07	0.432	−0.11/0.25	−0.03	0.756	−0.24/0.18	−0.04	0.677	−0.21/0.14	−0.12	0.192	−0.30/0.06	−0.05	0.680	−0.27/0.18	−0.04	0.630	−0.22/0.10
BRIEF-BRI	0.16	0.234	−0.11/0.42	0.33	0.032	0.03/0.64	0.26	0.041	0.01/0.51	−0.03	0.842	−0.29/0.24	0.29	0.080	−0.04/0.61	0.24	0.067	−0.17/0.50
BRIEF-MI	0.37	0.044	0.01/0.72	0.51	0.016	0.10/0.92	0.48	0.006^*^	0.14/0.81	0.53	0.004^*^	0.18/0.88	0.68	0.002^*^	0.25/1.12	0.62	0.001^*^	0.27/0.97
Model's *R*^2^	0.277			0.367			0.391			0.247			0.372			0.440		
Model's *p*-value	<0.0001^*^			<0.0001^*^			<0.0001^*^			<0.0001^*^			<0.0001^*^			<0.0001^*^		

* *p < 0.01 (2-tailed)*.

*B, Unstandardized Beta coefficients; Sig., significant level; 95%, confidence interval for B for each factor entered in the regression models with measure of the Social Responsiveness Scale as outcome*.

## Discussion

### The importance of the metacognition index

The main finding of the present study was that the metacognitive component of EF (MI), was the most important factor in explaining social function in children with ASD. We hypothesized that both BRI and MI from the BRIEF would predict SRS scores in children with ASD. However, despite high BRI scores, the MI explained more of the social dysfunctions measured with SRS in children with ASD. This is an interesting finding, since other studies have shown that behavior regulation is closely linked to social function (Kenworthy et al., 2009), and that metacognition competence is of more importance for school performance (Carretti et al., 2014). MI is composed of subdomains like initiating, working memory, organizing and monitoring. Difficulties in these areas are probably easier to overlook by parents, teachers and clinicians in everyday life, than difficulties with subdomains within behavior regulation like; inhibition, flexibility, and emotional control. Therefore, it is important to highlight that MI is of importance for social function. The current findings provide new knowledge about the relationship between EF and the various domains of social competence in children with ASD, using parent-rated measures.

There are few studies regarding the relationship between EF in everyday life settings and social function in children with ASD. The current findings are in line with Leung et al. (2015), who showed that the MI from the BRIEF plays a role for social function in children with ASD. However, in contrast to the finding from Leung et al. (2015), our study did not find a statistically significant contribution of the BRI to social function. There is no obvious reason for these different findings, and the participants in the two studies share many of the same characteristics. However, some differences in the recruitment process or sample size may explain the different results, and further studies are needed to clarify this question. Our study contained a larger number of participants and had a more conservative significant level compared to Leungs study, and a proportion of our participants also had comorbid ADHD, and this may explain the different results. However, the results from both studies emphasize the importance of the relationships between MI and social function in children and adolescents with ASD. Kenworthy et al. (2009), on the other hand, found that EF such as BRI predicted only the communication symptoms and not the reciprocal social interaction in children with ASD. However, they used a composite score based on ADI and ADOS scores to measure social function, and not the SRS (Kenworthy et al., 2009). Landa and Goldberg (2005) found no relation between the neuropsychological assessments of EF and the social function in ASD children. The difference to our findings may be a result of biases in parent rated approaches (e.g., an inclination to score “favorable” or “unfavorable” to items, regardless of the specific content), but it is also reasonable to assume that a parent-rated design may uncover some relevant aspects of everyday function not accessible to the controlled setting of standardized assessments.

We did not find any significant differences between girls and boys in our sample. However, girls had a tendency for higher scores on especially SRS total and Social Cognition, which might imply that girls have more social problems than boys in our sample. Contrary to White et al. (2017), the girls in our sample did not have significantly higher scores on the BRIEF than the boys. Others have found that females with ASD have more EF impairments compared to males (Lemon et al., 2011), while Lehnhardt et al. (2016) and Bolte et al. (2011) found evidence for higher EF functioning for females with ASD than for males. Sex did not have an independent impact in our regression models, but showed strong tendencies toward significance on the SRS total score and the subscale Social Cognition, where girls had more problems than boys in our sample. None of the studies earlier mentioned have investigated the relationship between EF and social function in everyday life settings, but it underlines the importance of being aware of possible sex differences in ASD.

Even though we did not find any significant differences between the two age groups children (6–12 years) and adolescents (13–18 year), there were some interesting tendencies. The BRIEF scores in our sample were quite similar in the two age groups, which is in contrast to Rosenthal et al. (2013) who found greater EF problems especially in metacognition for older children with ASD. Our result, that the relationship between EF difficulties and social dysfunction was strongest in the youngest group, might be due to young children having more generalized difficulties in both social function and EF than older children. However, we found that in the oldest group there was a significant relationship between social function and metacognition, but not for social function and behavior regulation. This might imply that metacognitive EF is of importance for social function in older age, and that behavior regulation does not have the same impact on social function. In our regression analyses we showed that age significantly predicted the ability to perceive social cues (Social Awareness), were the youngest group had more problems than the oldest group.

Intelligence (IQ level) did not have any significant impact on the relationship between EF and social function in our analysis. The relationship between measurements of EF, and especially fluid IQ, has been the subject of a longstanding discussion. Several studies have found that much of the variance in EF performance can be explained by IQ level (Joseph and Tager-Flusberg, 2004; Friedman et al., 2006; Diamond, 2013). However, there is indication that the relationship between EF and IQ is different among individuals with ASD, compared with typically developed (TD) and other patients groups. Merchan-Naranjo et al. (2016) found that EF was affected, but did not correlate with IQ in children and adolescents with ASD without intellectual disability. Rommelse et al. (2015) even found that participants with above average intelligence performed relatively more poorly on some EF tasks compared to IQ matched controls. One possible explanation for the different relationship between EF and IQ in individuals with ASD might be the role of speed of information processing. This ability can be intact in ASD, and not correlated to IQ. However, speed of information processing is normally closely linked to the general factor (g-factor) of intelligence (Scheuffgen et al., 2000; Wallace et al., 2009). The g-factor was established by Spearman, who discovered that most cognitive tests are positively correlated with each other, regardless of which cognitive domain the individual test measures (Colom et al., 2006). A review by Sheppard and Vernon (2008) concluded that measures of mental speed and information processing are significantly correlated with measures of intelligence in non-clinical samples. This highlights the importance of incorporating IQ as a covariant in the relationship between the EF and social function.

Earlier research has shown that TD children have a different relationship between EF and social function than children with ASD. In TD children, the BRI has an impact on their social function (Leung et al., 2015). Furthermore, there is evidence suggesting that there are mutual interactions between EF and social function for both TD and ASD groups (Moriguchi, 2014; Leung et al., 2015). From studies of TD children, we know that social interaction may facilitate the development of EF. Especially maternal scaffolding, modeling and imitation have demonstrated to be predictors of the development of EF (Moriguchi, 2014). It is also likely that EF facilitates the development of cognitive skills that are important for social interaction (van Lier and Deater-Deckard, 2016). Subcomponents of EF, such as inhibition, flexibility and monitoring are thought to influence the ability to engage in positive social interactions (van Lier and Deater-Deckard, 2016). White (2013) offers a different interpretation of the difficulties that individuals with ASD experience on especially unstructured EF tasks. She argues that poor results on these tasks are caused by difficulties in forming an implicit understanding of the experimenter's expectations for the task. In this view, implicit information is less available to individuals with ASD due to their mentalizing difficulties, and this leads to poor performance, more than difficulties with EF *per se*.

### Potential implication for development of clinical interventions

Our finding that subdomains of social function have a different relation to EF may be relevant for stratifying the treatment for children with ASD. Interventions that target metacognitive skills have, in earlier studies, shown to improve the social abilities in children and adolescents with ASD (Kenworthy et al., 2014; Leung et al., 2015). However, as social ability is a broad concept comprising different abilities and motivational factors, more studies are needed to identify which part of social function may profit on metacognitive interventions. Our study indicates that the ability to perceive social cues and social motivation has, possibly, a weaker relationship to EF than the other aspects of social functioning. This might imply that children with problems related to social communication, social mannerisms and social cognition might benefit more from intervention programs designed to enhance EF in general, and metacognitive function, in particular. Individuals that primarily have problems with the ability to pick up on social cues and social motivation might be less likely to benefit from such intervention programs. Taken together, it is possible to hypothesize that children with difficulties with understanding social cues (social awareness) might benefit more from classic social skills training programs (Chang et al., 2014; Otero et al., 2015). For children with problems related to social motivation it might be important to build on the child's areas of interest to enhance their motivation (Dunst et al., 2011). In all cases, we need to assess the individual child's cognitive and social profile and then tailor interventions to fit the child. Further studies are needed to examine the clinical implications of these findings. More generally, the finding that there is an important relationship between EF and social function, gives support to the hypothesis that an intervention that has an impact on cognitive abilities and EF is also likely to have an influence on the social skills of children and adolescents with ASD (McGovern and Sigman, 2005; Wang et al., 2017).

### Strengths and limitations of the study

The strengths of the study include a reasonable sample size of clinically well-defined children and adolescents with ASD, with a moderate sample of females. Both the SRS and the BRIEF are parental ratings, and this might bias the findings. However, we considered that parents observe their children when they are engaged in a range of everyday activities and that their observations add valuable information. Particularly since it is known that difficulties may not be observable in a laboratory setting or structured clinical settings, informant measures might have higher ecological validity (Kenworthy et al., 2008). At the same time, parent-ratings are likely to be affected by other factors such as IQ, emotional/behavioral problems and comorbidities in the children (Aldridge et al., 2012; Cholemkery et al., 2014; Havdahl et al., 2016). However, diagnostic tools like the ADOS and the ADI-R are also influenced by these kinds of confounding factors (Havdahl et al., 2016). Some of the children in our study were medicated, and several had a comorbid disorder. It is known from the literature that comorbidity is common in ASD, and that as many as 70% have a comorbid disorder (Simonoff et al., 2008; Lai et al., 2014; Gjevik et al., 2015). The main finding that metacognitive aspects of EF are significantly related to social function was significant also after removing the children with comorbid ADHD from the analysis. It can be argued that it is important to study individuals with ASD and comorbid ADHD because this is a common comorbidity. A recent meta-analysis of EF and children and adolescents with high-functioning ASD, was the first to take the confounding effect of ADHD comorbidity into account. They confirmed the presence of executive dysfunction in this group, and found that these deficits were not solely accounted for by the effect of comorbid ADHD or general cognitive abilities (Lai et al., 2016). Furthermore, language deficits are important factors that affect social communication/function in a negative way in ASD. We did not perform a comprehensive assessment of different language functions in our participants, but some studies have found that EF is not related to language ability in verbal, school-aged children with ASD (Joseph et al., 2005). Since our participants were school-aged children within the normal IQ range, we assume that our finding is valid, even without a comprehensive language assessment. The current sample included a wide age range and we therefore controlled for this in the regression analyses. Despite this, the age range might be a methodological limitation due to the correlation with social awareness, and future studies might benefit from focus on more restricted age groups.

Our study did not clarify in which direction EF and social function affect each other, but it suggests that there is a strong relation between the two. The current findings should be replicated in independent samples and combined with objective measurements, such as neuropsychological or neurophysiological examination. This was not available in the current sample. Thus, future research should combine laboratory and informant-based measures for a more in-depth investigation of the link between EF and social function, as the two measures are complementary to each other (Leung et al., 2015). To fully understand the relationship between EF and social functioning, studies with longitudinal designs are need to provide more specific detail about functional developmental change at different ages (Taylor et al., 2015). Furthermore, most research within the ASD field is conducted on male samples, but some evidence suggests that females exhibit greater cognitive impairments than their male counterparts (Lemon et al., 2011; White et al., 2017). In our analysis, we found a tendency for girls to have higher SRS scores, and a stronger relationship between social function and EF than boys. For future research, it is important to investigate if the relationship between EF and social function can be modulated by sex.

## Conclusion

We report a relationship between parental reports of EF and social function in an everyday setting in children with ASD. We found that the metacognitive domain of EF has a significant association to many aspects of social function. These results may have implications for understanding the cognitive components in the social deficits that define ASD. Further studies are needed to clarify if children with ASD will improve their social function through intervention programs designed to enhance EF in general and metacognitive function, in particular.

## Author contributions

Conceived and designed the study: TT, TN, MØ, and OA. Performed the study: TT, TN, and NS. Analyzed the data: TT and TN. Interpreted the data, wrote the paper, and approved the version to be published: TT, TN, MØ, NS, and OA.

### Conflict of interest statement

The authors declare that the research was conducted in the absence of any commercial or financial relationships that could be construed as a potential conflict of interest.
